# Twentieth Century Practice

**Published:** 1897-12

**Authors:** 


TWENTIETH CENTURY PRACTICE. An International Encyclopedia of Modern Medical Science. By leading authorities of Europe and America. Edited by THOMAS L. STEDMAN, M. D., New York City. In twenty volumes. Volume XII., Mental Diseases, Childhood and Old Age. New York: William Wood & Company. 1897.

This book is entirely European in its contributions. The section of mental disease is written by G. Fielding Blandford.

We have read this section for the purpose of ascertaining his views on questions still in dispute—and not with the idea of reading his accurate descriptions of the well-known types of insanity and their treatment. We find he admits the fact of partial insanity, for he says: "These partially insane folk arrive at the point of delusion in one of two ways."

He classifies these patients as monomaniacs and divides them into monomania of pride, of jealousy, of hoarding, of persecution and of doubt. We do not consider his view as one that will meet with much favor in this country. In the United States the tendency is to deny to monomania a place in classification. We do this because the term is misleading. Such patients rarely are maniacal. Stearns more nearly represented the American view on this question when he said: "I think there can be no question that it is neither single in its elements nor maniacal in its manifestations and that the term monomania is, therefore, untrue in both its primal and terminal compositions and thus vitiates all principles of medical nomenclature."

Of moral insanity he says, and we believe most thoroughly with him, "The stage of moral insanity is one on the way to mania with delusions and sometimes a stage on the way to general paralysis." He denies the proposition that morality is a division of the mind apart from intellect. Therefore, we cannot have a moral insanity with a sound intellect. Such a statement, he truly says, is "a quibble about words."

Concerning the question, "Was the person whose act is in question able to understand its nature and to pass a fairly rational judgment on its consequence to himself and others and was he a free agent so far as that act was concerned ?" Blandford states, "That this is the test which medical men would lay down may be assumed without hesitation." Krafft-Ebing's Psychopathia Sexualis finds no advocate in Blandford.

We are pleased to notice a reference to the late Dr. J. B. Andrews.

Idiocy is considered in 110 pages by Paul Sollier, of Paris. He considers Bourneville's classification as the most complete.

The description of various idiots, their physical and mental condition, are well drawn. The practical portion and the most interesting to the general practitioner is the discussion of treatment of Lannelongue's operation. He says: "This operation, based on pathological, pathogenic, physiological and even mechanical error, was soon entirely abandoned." The medico-pedagogic treatment of Bourneville is well described and has his indorsement.

Lombroso discusses criminal anthropology in condensed form. The article is illustrated with portraits of types of criminals. He discusses fully the therapeutics of crime. His bibliography contains but one reference to an American.

Boy-Tessier, of Marseilles, is allotted 105 pages to discuss the diseases of old age. He considers (1) normal senility; (2) the diseases of senility; (3) the modifications in ordinary disease as observed in the aged. Of Brown-Sequard's injections of testicular juice he says: "I regard this agent as of real power." The section is not one of great value, as it omits too much.

Diseases of children, by Jules Comby, of Paris, occupies about 300 pages. This section includes all the children's diseases except infectious diseases and rachitis. The various affections are treated in condensed style and include the usual discussion of such subjects.

We notice with pardonable pride a reference to Dr. Krauss's article on Incontinence of urine, published in BUFFALO MEDICAL JOURNAL, 1891. The volume is fully up to the standard of others in the series. J. W. P.

